# The 5-HT_1F_ receptor agonist lasmiditan as a potential treatment of migraine attacks: a review of two placebo-controlled phase II trials

**DOI:** 10.1007/s10194-012-0428-7

**Published:** 2012-03-20

**Authors:** Peer C. Tfelt-Hansen, Jes Olesen

**Affiliations:** Department of Neurology, Danish Headache Center, Glostrup Hospital, University of Copenhagen, Glostrup, Denmark

**Keywords:** Lasmiditan, 5-HT_1F_ receptor agonism, Migraine, Randomised controlled trial, Phase II

## Abstract

Lasmiditan is a novel selective 5-HT_1F_ receptor agonist. It is both scientifically and clinically relevant to review whether a 5-HT_1F_ receptor agonist is effective in the acute treatment of migraine. Two RCTs in the phase II development of lasmiditan was reviewed. In the intravenous placebo-controlled RCT, lasmiditan doses of 2.5–45 mg were used, and there was a linear association between headache relief (HR) rates and dose levels (*P* < 0.02). For lasmiditan 20 mg, HR was 64 % and for placebo it was 45 % (NS). In the oral placebo-controlled RCT, lasmiditan doses of 50, 100, 200 and 400 mg were used. For HR, all doses of lasmiditan were superior to placebo (*P* < 0.05). For lasmiditan 400 mg, HR was 64 % and it was 25 % for placebo. Adverse events (AEs) emerging from the treatment were reported by 22 % of the patients receiving placebo and by 65, 73, 87 and 87 % of patients receiving 50, 100, 200 and 400 mg, respectively. The majority of AEs after lasmiditan 100 and 400 mg were moderate or severe. For the understanding of migraine pathophysiology, it is very important to note that a selective 5-HT_1F_ receptor agonist like lasmiditan is effective in the acute treatment of migraine. Thus, migraine can be treated with a drug that has no vasoconstrictor ability. While lasmiditan most likely is effective in the treatment of migraine attacks it had, unfortunately, a high incidence of CNS related AEs in the oral RCT. If confirmed in larger studies in phase III, this might adversely limit the use of this highly specific non-vascular acute treatment of migraine. Larger studies including the parameters of patients’ preferences are necessary to accurately position this new treatment principle in relation to the triptans.

## Introduction

The pathophysiology of migraine is incompletely understood. Previously, extracranial dilatation was considered pivotal in causing migraine headache [1]. The selective 5-HT_1B/1D_ receptor agonists, triptans, were developed as relatively selective cranial vasoconstrictors based on the efficacy on the 5-HT_1B_ receptor [2, 3]. This receptor is also present in non-cranial vasculature [4, 5] and the triptans carry the risk of causing coronary vasoconstriction [4]. The triptans are thus contraindicated in patients with cardio- and cerebrovascular disease. The CGRP antagonists, olcegepant [6], telcagepant [7], BI 44370 TA [8] and MK-3207 [9] were developed for migraine as drugs devoid of general vasoconstrictor activity [10]. They were effective in the acute treatment of migraine [6, 7], but the developments were stopped for various reasons [11].

It has been suggested that cranial vasodilatation, observed previously [12] and quite recently [13], is not the primary nociceptive stimulus for migraine headache [14, 15], and that neural inhibition of trigeminal pathways could provide an alternative non-vascular antimigraine mechanism [15].

## Preclinical pharmacological profile of lasmiditan [16]

In vitro binding studies of lasmiditan showed a *K*_i_ value of 2.2 nM at the 5-HT_1F_ receptor, compared with *K*_i_ values of 1,043 and 1,357 nM at the 5-HT_1B_ and 5-HT_1D_ receptors, respectively, which is a selectivity ratio >470-fold [16]. Unlike sumatriptan, a 5-HT_1B/1D_ receptor agonist, lasmiditan did not contract rabbit saphenous vein rings at concentrations up to 100 μM [16]. In two rodents models with presumed relevance for migraine (dural plasma extravasation, and induction of the immediate early gene *c*-*Fos* in the trigeminal nucleus caudalis), oral administration of lasmiditan potently inhibited these markers associated with electrical stimulation of the trigeminal ganglion [16]. The oral bioavailability of lasmiditan is 40 % and the *T*_max_ is 2 h (CoLucid Pharmaceuticals, data on file).

## Review of phase II trials

Lasmiditan has so far been investigated in two RCTs: one with intravenous [15], and one with oral administration [17] of the drug.

The intravenous RCT was a randomised, multicentre, double-blind, placebo-controlled, group-sequential, adaptive treatment-assignment, proof-of-concept and dose-finding study [15]. The investigators treated 130 in-hospital migraine patients with moderate or severe headache. The patients were allocated to a range of intravenous dose levels of lasmiditan or placebo in small cohorts (*n* = 5–6). The starting dose was 2.5 mg. Subsequent doses were adjusted up and down according to the efficacy and safety observed in the preceding cohort. The primary efficacy measure was headache relief (a decrease in headache from moderate or severe to none or mild) at 2 h.

**Fig. 1:**
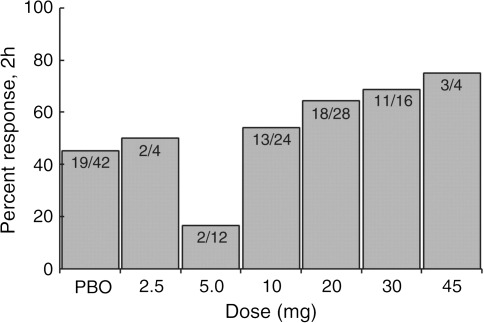
Proportion of migraine patients with headache relief (a decrease of headache from moderate or severe to none or mild) (HR) at 2 h after intravenous lasmiditan (*PBO* placebo) [15]

A total of 88 patients received lasmiditan in doses of 2.5–45 mg, and 42 received placebo. The study was terminated when the 20 mg dose met predefined efficacy stopping rules [15]. As illustrated in Fig. 1 there was a linear association between response rates and dose level (*P* = 0.0126) [15]. For lasmiditan 20 mg, the headache relief was 64 % and for placebo it was 45 %. Thus, the therapeutic gain (percentage difference between active drug and placebo) was 19 % (95 % CI −4 to 42 %). Adverse events were generally mild and were reported by 65 % of patients on lasmiditan and 43 % on placebo [15].

**Fig. 2:**
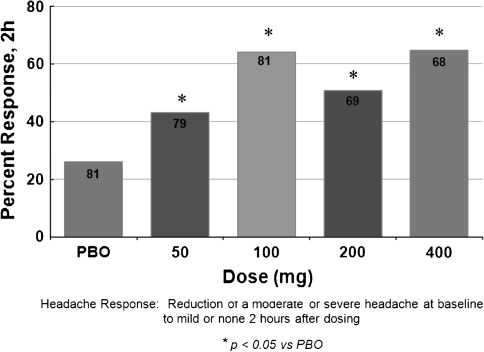
Proportion of migraine patients with HR at 2 h after oral lasmiditan 50–400 mg (*PBO* placebo). *P < 0.05 [17]

The oral RCT with lasmiditan was a randomised, double-blind, placebo-controlled, parallel-group study [17]. Patients were randomized to oral lasmiditan (50, 100, 200 or 400 mg) or placebo in a 1:1:1:1.1 ratio. Out of 534 screened and randomized patients, 391 treated a migraine attack and 378 patients qualified for the primary modified intent-to-treat analysis [17]. Patients treated moderate or severe migraine headache, and the primary efficacy measure was headache relief (HR) 2 h after drug administration (see Fig. 2; Table 1). Headache response for all doses of lasmiditan was superior to placebo (*P* < 0.05) (Fig. 2). For lasmiditan 400 mg, the therapeutic gain was 38 % (95 % CI 28–51 %) (Table 1). Adverse events emerging from the treatment were reported by 22 % of the patients receiving placebo and by 65, 73, 87 and 87 % of patients receiving 50, 100, 200 and 400 mg lasmiditan, respectively [17]. The AEs for placebo and lasmiditan 100 and 400 mg are shown in Table 2. The distribution of intensity of AEs after placebo and oral lasmiditan 100 and 400 mg is shown in Table 3. The majority of AEs after placebo were mild (15 %) or moderate (13 %), whereas the majority of AEs after lasmiditan were moderate [46 % (100 mg), 60 % (400 mg)] or severe [27 % (100 mg), 44 % (400 mg)] [CoLucid Pharmaceuticals, data on file].

**Table 1:** Headache relief after intravenous and oral lasmiditan, subcutaneous and oral sumatriptan, and oral LY334370 in randomised, clinical trials (RCTs) [7, 15, 17, 20]

Drug	Headache relief for active drug at 2 h (%)	Headache relief for placebo at 2 h (%)	Therapeutic gain (95 % confidence intervals) (%)	NNT (number needed to treat)
Intravenous lasmiditan 20 mg [15]	64	45	19 (−4 to 42 %)^a^	5.3
Oral lasmiditan 400 mg [17]	64	25	38 (28 to 51 %)	2.6
Subcutaneous sumatriptan 6 mg [7]	69	19	51 (48 to 53 %)	2.0
Oral sumatriptan 100 mg [7]	61	28	33 (31 to 35 %)	3.0
LY334370 200 mg [20]	71	19	52 (27 to 77 %)	2.0

^a^The RCT did not have the power to compare the single doses of lasmiditan with placebo

**Table 2:** Adverse events with an incidence >5 % of patients in any dose group [17]

Adverse event	Placebo (%)	Lasmiditan 100 mg (%)	Lasmiditan 400 mg (%)
Dizziness	1	28	37
Fatigue	2	21	24
Vertigo	1	15	24
Somnolence	2	12	11
Paraesthesia	2	11	20
Heaviness	1	5	7
Nausea	0	11	7

**Table 3:** Adverse events by intensity after placebo and oral lasmiditan 100 and 400 mg (CoLucid Pharmaceuticals, data on file)

Intensity of adverse events	Placebo (*n* = 86)	Lasmiditan 100 mg (*n* = 82)	Lasmiditan 400 mg (*n* = 70)
Mild	14 (16 %)	35 (43 %)	21(30 %)
Moderate	11 (13 %)	38 (46 %)	42 (60 %)
Severe	5 (6 %)	22 (27 %)	31 (44 %)

## Discussion

The intravenous randomised controlled trial of lasmiditan [15] should be considered as a proof-of-concept study validating the principle of 5-HT_1F_ receptor agonism in the acute treatment of migraine. It was not powered to demonstrate superiority of the individual doses of lasmiditan to placebo (see Fig. 1). The oral study [17] documented beyond doubt that 5-HT_1F_ agonism is highly effective (Fig. 2), perhaps as effective as the triptans. Usually one would expect an intravenous administration of a drug to be more effective and cause more adverse events (AEs) than the oral form of the drug. With lasmiditan, the case was the opposite: oral administration is better than the intravenous administration as judged from the therapeutic gains which were 38 and 19 %, respectively (Table 1). The reason for these results is most likely a relatively low intravenous dose of 20 mg lasmiditan. The oral dose of lasmiditan was 400 mg and the oral bioavailability of lasmiditan is 40 % (CoLucid Pharmaceuticals, data on file). Thus, an oral dose of 400 mg corresponds to an intravenous dose of 160 mg far above the doses (2.5–45 mg) used in the intravenous RCTs. A high placebo response with intravenous treatment may, however, also diminish the TG. Therefore, the absolute response is also important and for oral lasmiditan it was similar to previous results with oral triptans.

Adverse events should also be evaluated by their absolute size and by subtracting the AEs after placebo from AEs after active drug. For oral lasmiditan 400 mg, the placebo-subtracted AEs rate is 62 % [number needed to harm (NNH) 1.6]. For intravenous lasmiditan 20 mg the placebo-subtracted AEs is 25 % (NNH 4). Thus, oral lasmiditan 400 mg caused more AEs than the intravenous dose of 20 mg as would be expected from the higher dose absorbed with the oral 400 mg dose. For the standard triptan, sumatriptan 100 mg, the placebo-subtracted AEs rate is 16 % (NNH 6.3) [18].

The high incidence of AEs for lasmiditan is of potential concern, but needs further evaluation due to small numbers. In future RCTs, one should include also a global evaluation of study medication, such as excellent, very good, good, neither good nor bad, poor, very poor and extremely poor [8]. This would allow an estimation of how patients really rate the recorded adverse events.

The results seem to suggest that a dose of 100 mg might be preferable to 400 mg because apparently it had the same efficacy in terms of headache relief (see Fig. 2). This would, however, only result in a minor decrease in AEs to 73 %. Paradoxically, the pain-free response at 2 h was considerably smaller with 100 than with 400 mg. If this is real and not just due to statistical fluctuation, pain-free response would decrease from 28 (lasmiditan 400 mg) to 14 % (lasmiditan 100 mg) [17]. Migraine patients want to be pain free [19] and 14 % pain free is too low for a drug to be successful in our opinion. Again, more studies with higher numbers are necessary to answer these questions.

The effect of intravenous lasmiditan should be compared to the current standard triptan, sumatriptan, as illustrated in Table 1. The therapeutic gain (TG) (percentage difference between active drug and placebo) for intravenous lasmiditan 20 mg was 19 % (95 % CI −4 to 42 %) and it was 51 % (95 % CI 48–53 %) for subcutaneous sumatriptan 6 mg [18]. This lower response for intravenous lasmiditan compared with subcutaneous sumatriptan probably reflects the low dose of intravenous lasmiditan.

The oral administration of lasmiditan 400 mg should be compared to the standard triptan, sumatriptan 100 mg, and the previously investigated 5-HT_1F_ receptor agonist LY334370 200 mg [19]. The TG for lasmiditan (*n* = 156) was 38 % (95 % CI 28–51 %) comparable to the TG of 33 % (95 % CI 31–35 %) for oral sumatriptan (*n* = 5,072) [18]. The TG for 5-HT_1F_ receptor agonist LY334370 200 mg (*n* = 47) was 52 % (95 % CI 27–77 %) (Table 1) [20] and apparently higher than the two other oral drugs. However, as few patients were included in RCTs of lasmiditan and LY334370 resulted in very wide confidence intervals; therefore, superiority as compared with sumatriptan should not be claimed.

For the understanding of migraine pathophysiology, it is very important to note that a selective 5-HT_1F_ receptor agonist like lasmiditan is effective in the acute treatment of migraine. This is supported by the previous results with the other 5-HT_1F_ receptor agonist, LY333470 [20] (see Table 1). Thus, migraine attacks can be treated with a drug that has no vasoconstrictor ability (it remains to be seen if vasoconstriction of vessels dilated because of a migraine attack does occur). The 5-HT_1F_ effect may take place at the perivascular trigeminal nerve terminals, which will be stabilized and less likely to leak vasoactive and potentially nociceptive signalling molecules. However, Burstein’s group has shown that this mechanism is unimportant with the triptans which rather seem to work by blocking nociceptive transmission at the first synapse in the trigeminal nucleus caudalis [21]. It seems likely that the same is the case for 5-HT_1F_ receptor agonists. Seemingly, this would support the neural theory of migraine [14]. However, blocking the trigeminovascular system would also be effective if peripheral nociception was the primary cause of headache [22].

In conclusion, the 5-HT_1F_ receptor agonist lasmiditan is effective in the acute treatment of migraine. Unfortunately, it has a high incidence of CNS-related side effects. If confirmed in larger studies, this might adversely affect the uptake of this highly specific non-vascular acute treatment. Larger studies including the parameters of patients’ preferences are necessary to accurately position this new treatment principle in relation to the triptans.
